# Establish axenic cultures of armored and unarmored marine dinoflagellate species using density separation, antibacterial treatments and stepwise dilution selection

**DOI:** 10.1038/s41598-020-80638-x

**Published:** 2021-01-08

**Authors:** Thomas Chun-Hung Lee, Ping-Lung Chan, Nora Fung-Yee Tam, Steven Jing-Liang Xu, Fred Wang-Fat Lee

**Affiliations:** 1 Department of Science, School of Science and Technology, The Open University of Hong Kong, Ho Man Tin, Hong Kong; 2 Department of Chemistry, City University of Hong Kong, Kowloon Bay, Hong Kong

**Keywords:** Biological techniques, Ecology, Environmental sciences, Ocean sciences

## Abstract

Academic research on dinoflagellate, the primary causative agent of harmful algal blooms (HABs), is often hindered by the coexistence with bacteria in laboratory cultures. The development of axenic dinoflagellate cultures is challenging and no universally accepted method suit for different algal species. In this study, we demonstrated a promising approach combined density gradient centrifugation, antibiotic treatment, and serial dilution to generate axenic cultures of *Karenia mikimotoi* (KMHK). Density gradient centrifugation and antibiotic treatments reduced the bacterial population from 5.79 ± 0.22 log_10_ CFU/mL to 1.13 ± 0.07 log_10_ CFU/mL. The treated KMHK cells were rendered axenic through serial dilution, and algal cells in different dilutions with the absence of unculturable bacteria were isolated. Axenicity was verified through bacterial (16S) and fungal internal transcribed spacer (ITS) sequencing and DAPI epifluorescence microscopy. Axenic KMHK culture regrew from 1000 to 9408 cells/mL in 7 days, comparable with a normal culture. The established methodology was validated with other dinoflagellate, *Alexandrium tamarense* (AT6) and successfully obtained the axenic culture. The axenic status of both cultures was maintained more than 30 generations without antibiotics. This efficient, straightforward and inexpensive approach suits for both armored and unarmored dinoflagellate species.

## Introduction

The frequency of harmful algal blooms (HABs) has been increasing worldwide over the past decades, which have major economic effects on fish farming, the shellfish industry, as well as human health^1,2^. Dinoflagellates, major causative agents of HABs, include *Karenia mikimotoi* and *Alexandrium tamarense*, are highly toxic. *K. mikimotoi* and *A. tamarense* blooms are frequently associated with mass killings of fish, shellfish, and marine invertebrates^3,4^. Control of HABs is imperative and research on HAB-causing dinoflagellate species is therefore crucial.

Similar to other microalgal species, dinoflagellates typically contain a substantial amount of bacteria in the laboratory cultures^5^. The associated bacteria can naturally attached onto the algal cell surface and coexist in the free-living form in the algal culture^6^, thus interfering the consortia^7^. The growth and toxicity of the axenic and xenic cultures of the same microalgal species differ significantly^8^. Complicated consortia create additional difficulties in the investigation of microalgae–bacteria interactions. For the control of HABs, the use of bacteria as an algicidal agent has been studied extensively^9^. Such studies often require the isolation of clonal microalgal cultures and the cultivation of a reliable axenic culture of the target algal species. Axenic algal cultures are also necessary for commercial applications of microalgae biomass^10^ and even molecular genetic research, such as DNA sequencing^11^. Contaminated sequences generated from xenic cultures during genome sequencing induce noise and increase the complexity during bioinformatics analysis^11^. It is crucial to secure axenic microalgal culture for various aspects of algal research^11^.

An axenic culture is a culture of a single strain or species without any other contaminating organism. There is no universal method for obtaining axenic cultures from different microalgal species. Various methods have been proposed for developing axenic cultures of microalgae, but only a few were for dinoflagellate species because of the fragile structure of unarmed species and difficulty in completely removing the bacteria attached onto cell surface^12–14^. Antibiotics are commonly used in the development and maintenance of axenic dinoflagellate cultures^15,16^, but also cause negative effects on the algal cells^17,18^. More, the maintenance of axenic cultures involves repeated sub-culturing and lengthy experiments, making this antibiotics method ineffective and time-consuming. The micro-pipetting method is used to obtain an axenic culture of centric diatom, *Coscinodiscus wailesii*^19^, but the process is labor and skill intensive. Ki and Han successfully developed axenic cultures of two armored dinoflagellate species, *A. tamarense* and *Peridinium bipes*, by using the combination of filtration and algal phototaxis^12^. Yim and Lee also established an axenic *Gyrodinium impudicum* by using the combination of phototaxis and selection of axenic culture in 24 well plate^14^. Phototaxis is applicable only to phototactic algae. Some species, such as benthic and epiphytic *Prorocentrum lima*, may not exhibit a strong phototactic response, and the presence of other phototactic species in the mixture would also reduce the efficacy of phototaxis^20^. More, bacteria attached firmly onto the algal surface cannot be removed efficiently with algal phototaxis alone^12^. Another group of researchers developed an axenic culture of *A. tamarense* by treating the algal cells with lysozyme/SDS and antibiotics^13^. Their method is straightforward and efficient, but the lysozyme/SDS treatment can damage the dinoflagellate cells. Unarmored dinoflagellate species may particularly be sensitive to even a low-dose application of these bactericidal substances, and the method may be limited to only dinoflagellate species with a high tolerance to those bactericidal substances.

Most of the described methods, particularly those involving repeated centrifugation, filtration and bactericidal substances, may damage the unarmored dinoflagellate species such as *Cochlodinium* spp. and *Karenia* spp. due to the lack of protective cell walls or plates at the outermost layer of these cells. The present study aims to develop a method using a combination of three simple techniques, a series of Percoll density gradient centrifugation, antibiotic treatment and serial dilution selection for the establishment of an axenic dinoflagellate culture of *K. mikimotoi* (unarmored). The study also tested the efficacy and applicability of the developed method for another dinoflagellate species, *A. tamarense* (armored).

## Materials and methods

### Algal cultures and growth conditions

The unarmored dinoflagellate, *K. mikimotoi* used in the present study was isolated from an algal bloom occurred in Yim Tin Tsai of Tolo Harbor, Hong Kong in 2016 (denoted as KMHK) by our team. The armored species, *A. tamarense* was isolated locally and was provided by Prof. Samuel Lo (The Hong Kong Polytechnic University) in 2009 (denoted as AT6). Both cultures were maintained in the L1 medium^21^ without silicate. The salinity and pH of natural seawater, also collected from Tolo Harbor, were first adjusted to 30 ppt and 8, respectively, then filtered through a 0.45-μm membrane followed by autoclaving at 121 °C and 100 kPa for 20 min. Both algal cultures were maintained at 22 °C under a 12-h light–dark cycle of approximately 42 µmoles m^-2^ s^-1^ for 7 days to reach the log phase. Prior to adjustment of the initial cell density, the cell density of the algal culture was counted on a Sedgewick-Rafter cell counter under a light microscope (Optika, Italy) at 100× magnification.

### Discontinuous Percoll density gradient centrifugation

Artificial seawater was prepared by dissolving (0.015 g to 0.15 g) NaCl in 50 mL deionized water to different salinities (30–300 ppt), then sterilized using syringe filter (0.2 um). Percoll Plus (GE Healthcare, USA) was diluted using sterile seawater to achieve different concentrations, but the final salinity of each Percoll solution was maintained at 30 ppt. 90% Pecoll solution was added to a 15 mL centrifuge tube, overlaid with approximately 3.5 × 10^5^ dinoflagellate cells at the log phase, and centrifuged at 1000 rpm for 5 min without brake. Algal cells were then collected for subsequent rounds of centrifugation with different discontinuous gradients, including 90%, 90–50%, 90–50–30% and 90–50–30–10%. Prior to the next round of centrifugation, approximately 1 mL of the condensed algae was harvested and Percoll inside the algal sample was diluted using sterile seawater (30 ppt) to 5 mL before overlaid on the gradient. After all rounds of centrifugation were completed, the concentration of bacteria remained in the algal sample was determined using the spread plate technique on ST10^−1^ agar, containing 0.05 g of yeast extract, 0.5 g of trypticase peptone and 15 g of agar in 1 L of seawater^22^. The plate was incubated for 14 days at room temperature (22 °C) and the number of bacterial colonies on the agar was determined.

### Antibiotic susceptibility testing

Antibiotic susceptibility of algae-associated bacteria was determined by spreading 0.1 mL xenic KMHK culture on the same ST10^−1^ agar and incubated at the same condition as mentioned above, except the agar contained combinations of the following antibiotics: penicillin (100 U), streptomycin (100 µg/mL), gentamicin (100 µg/mL; Thermo Fisher, USA) and tetracycline (1 µg/mL; Sigma, USA). ST10^−1^ agar without antibiotics was used as the control. After 14-day of incubation, the number of bacterial colonies on the agar was recorded. The absence of bacterial colonies on the agar with antibiotics indicated bacterial susceptibility to the antibiotic cocktail.

### Basic and extended protocols

A series of steps comprising Percoll density gradient centrifugation and antibiotic treatments were separated into two experimental protocols: basic and extended (Supplementary Fig. 1). The basic protocol involved six steps: First, 3.5 × 10^5^ dinoflagellate cells were condensed through centrifugation with 90% Percoll (Step 1), and the algal sample was then centrifuged with a density gradient of 90–50–30% (the optimized gradient according to the result) two times (Steps 2 and 3). The algal cells were re-suspended into 10 mL of L1 medium and treated with mixed antibiotics for 48 h, and the algal sample was then centrifuged as Steps 1–3 again (Steps 4–6). The extended protocol was generally the same as the basic one, but had an additional incubation with mixed antibiotics for another 48 h and Steps 4–6 of the basic protocol were repeated. After each step of the basic and extended protocols, the total bacterial count in the algal sample was assessed as mentioned above. After the steps were completed, the algal cells obtained from basic and extended protocols were transferred into a 20-mL culture flask with an initial cell density adjusted to 1000 cells/mL, incubated at the same condition mentioned above for 7 days. The number of algal cells in the algal samples were counted at days 1, 3, and 7. The bacteria remained on algal sample were also determined simultaneously according to the method mentioned above.

**Figure 1:**
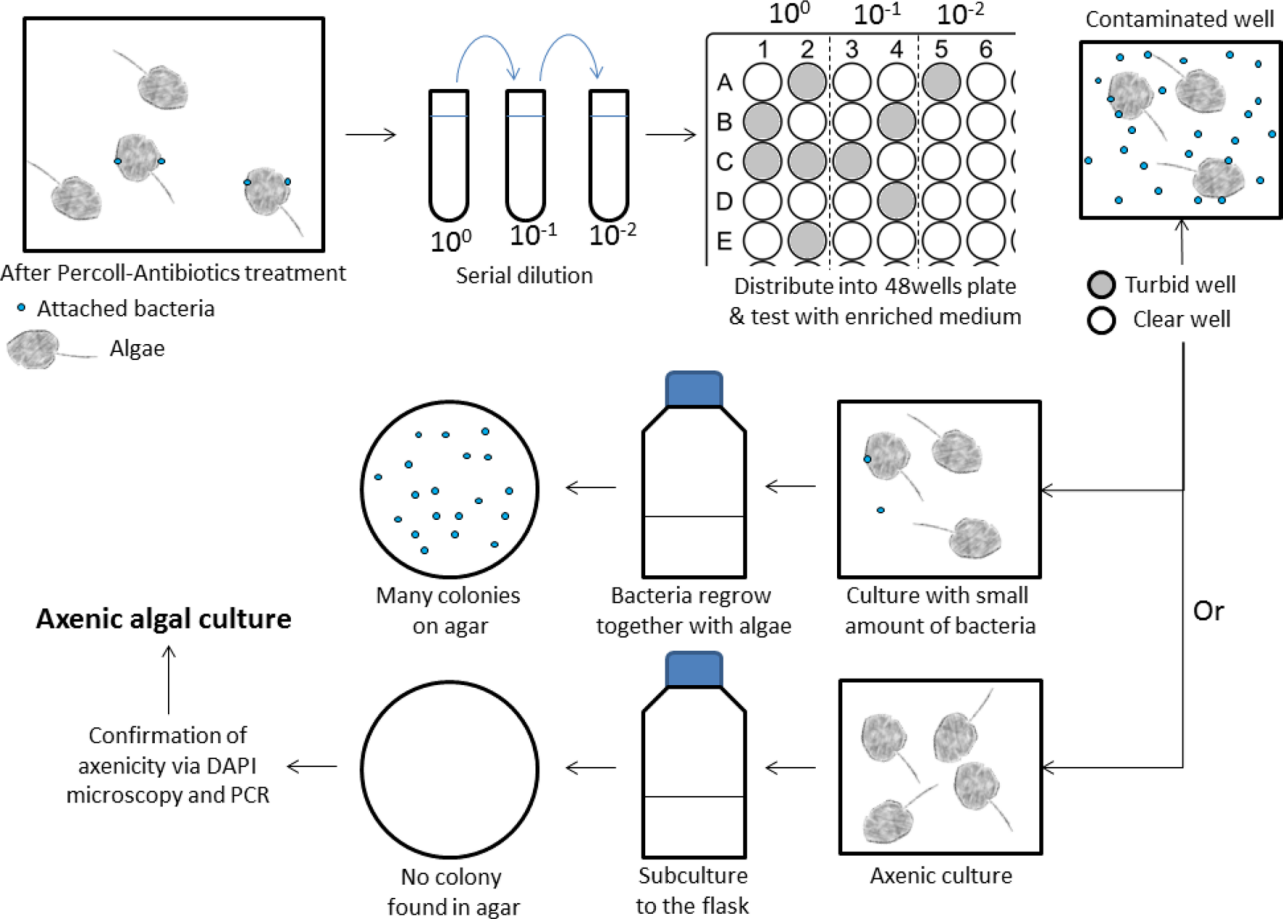
Selection of the axenic algal clones through serial dilution after the basic protocol.

### Effects of initial algal cell densities and antibiotic exposure times

The basic protocol was repeated using three initial algal cell number, 3.5 × 10^5^ cells (high), 1 × 10^5^ cells (moderate) and 3.5 × 10^4^ cells (low), and three antibiotic exposure times, 48, 72 and 96 h. The antibiotic exposure times were chosen based on our preliminary study that this algal species (KMHK) could tolerate the antibiotics up to 96 h. The total bacterial counts in the algal samples before and after the antibiotic treatment were recorded as mentioned above to calculate the amount and percentage of bacterial removal. Regrowth of bacteria in the treated algal samples at days 1, 3, and 7 under various conditions were assessed using the same plate count method described earlier.

### Axenic algal cells selection by serial dilution

After the basic protocol, algal cells collected were re-suspended into 10 mL of L1 medium and subjected to tenfold serial dilutions, 10^0^, 10^–1^ and 10^–2^ (Fig. 1). Next, 1 mL of algal cells in each dilution was evenly distributed into 10 wells of a 48-well plate, and 10 µL of the ST10^−1^ medium containing 0.05 g of yeast extract and 0.5 g of trypticase peptone in 1 L of seawater was added into each well. The algal cells were incubated for 7 days at the same conditions mentioned above. The growth of bacteria was promoted by adding 1% (v/v) ST10^−1^ medium in each well. The algae and bacteria grown in the wells were identified based on microscopic observation (including the morphology and motility of algae) and turbidity^13^, respectively. The wells that turned turbid indicating bacteria growth were discarded. In each dilution, at least five clear wells without turbidity and contained motile algal cells with unchanged morphology were randomly selected. Algal cells selected from individual wells were sub-cultured in a culture flask with 20 mL of L1 medium and cultivated under the same condition mentioned earlier. 100 µL of each algal culture was collected at days 7 and 21 for checking any bacterial contamination using the standard bacterial aerobic plate count method^23^. The basic protocol followed by the selection procedure was repeated three times, algal cultures without bacteria were then selected, The algal axenicity was further verified with DAPI epifluorescence microscopy, and bacterial 16 s ribosomal DNA (rDNA) and fungal internal transcribed spacer (ITS) sequence analyses.

### DAPI epifluorescence microscopy

DAPI epifluorescence microscopy was performed using Imai’s method^24,25^ with few modifications. In brief, 1 mL of algal cells was fixed with 1% glutaraldehyde and stained with DAPI (1 µg/mL; Thermo, USA) for at least 5 min. The stained sample was filtered through a sterile 0.2-μm black poretics polycarbonate track-etched membrane (GVS Life Science, USA). The sample was then transferred from the filter paper to a glass slide and observed under Nikon Eclipse Ts2R inverted fluorescence microscope (Nikon, Japan) at 1000× magnification.

### PCR amplification of bacterial 16 s rDNA and fungal ITS

The genomic DNA of the algal sample was extracted using the DNeasy Plant Pro Kit (Qiagen, Germany). The bacterial 16 s rDNA was amplified from the extracted DNA by using primers described previously^26^, 27F (5′-AGAGTTTGATCCTGGCTCAG-3′) and 1492R (5′-GGTTACCTTGTT ACGACTT-3′), whereas fungal ITS region was amplified using fungal ITS-specific primers: ITS1 (5′-TCCGTAGGTGAACCTGCGG-3′) and ITS4 primer (5′-TCCTCCGCTTATTGATATGC-3′)^27^. PCR was performed under the following conditions: 95 °C for 5 min, followed by 10 touchdown cycles of 94 °C for 40 s, 50 °C (-1 °C for each cycle) for 40 s, and 72 °C for 1 min, followed by 25 cycles of 94 °C for 40 s, 40 °C for 40 s, and 72 °C for 1 min and finally, 72 °C for 10 min. The PCR products were separated and visualized using 1% agarose gel electrophoresis. Target bands were cloned into pGEM-T easy vectors (Promega, USA) followed by DNA sequencing. DNA sequencing of all cloned plasmids was performed at commercial facilities using conventional dideoxy methodology.

### Statistical analysis

All data are presented as means ± standard derivations of three biological replicates. One-way analysis of variance (ANOVA) was used to determine any significant differences in bacterial concentration and algal regrowth between different steps in the basic and extended protocols. If the ANOVA is significant, Tukey multiple comparison test was used to find out where the difference lies. ANOVA was also employed to test differences in bacterial removal between initial algal cell densities and antibiotic treatment times.

## Results and discussion

### Percoll density gradient centrifugation

**Figure 2:**
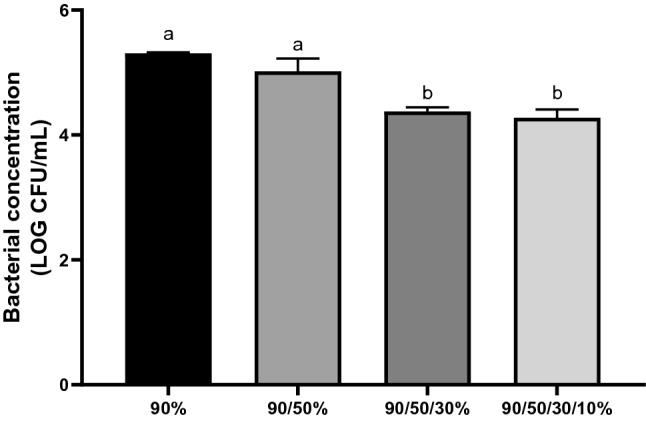
Total bacterial count in the algal sample after centrifugation with different Percoll density gradients. All data are presented as means ± standard deviations of three independent experiments (n = 3). Different letters on the top of the bar indicate that the means were significantly different among gradients at *p* ≤ 0.05 according to one-way analysis of variance followed by Tukey multiple comparison tests.

The removal of the associated bacteria from KMHK cells against four Percoll density gradients, 90%, 90–50%, 90–50–30% and 90–50–30–10% were shown in Fig. 2. There was no significant difference of the remaining total bacterial counts (with an initial total bacterial count of 6.36 ± 0.04 log_10_ CFU/mL) between algal samples centrifuged with 90% and 90%–50% Percoll gradients, but decreased significantly from 5.02 ± 0.2 log_10_ CFU/mL to 4.38 ± 0.05 log_10_ CFU/mL when dinoflagellate samples were centrifuged with 90–50–30% Percoll gradient with no further increase on adding another layer of 10% density to the gradient (i.e., 90–50–30–10%). These suggested that the highest bacterial removal capacity was achieved by centrifugation of the KMHK cells with the three-layer discontinuous (90–50–30%) gradient. This gradient was adopted in subsequent experiments in the present study, but it was different from Cho et al*.* who centrifuged the small Haptphyta, *Isochrysis galbana* (6–12 μm) with the five-layer discontinuous gradient (50%–40%–30%–20%–10%) and harvested the algal cell in between 40 and 30% Percoll^5,27^. As far as we know, this is the only previous study employing discontinuous gradient for algal culture, and it is obvious the optimized gradient composition varies among algal species, probably because of the diverse algal size and morphology. Vu et al. (2018) suggested that cells with a swimming ability may swim away from the concentrated zone after centrifugation, resulting in low cell recovery efficiency^11^. In this study, however, the swimming ability of KMHK cells was lost only temporarily for several minutes after centrifugation. This indicated that KMHK cell recovery would not be affected if the supernatant were removed immediately after centrifugation.

Density gradient centrifugation provides an excellent alternative to filtration and micro-pipetting to physically separate bacterial cells from the algal cells, especially for fragile cell separation^11^. The density medium provides padding and thus protects the algal cells from the shearing force and enhance the separation efficiency^17^. Although filtration is one of the commonly adopted separating techniques because of its convenience, inexpensive and easy to use^11^, it is infeasible for use with dinoflagellate samples. However, membrane filters can be easily clogged by the algal cells and thus prolong the processing time of the filtration. Our previous experience found that more than 1 h was required to filter 10 mL of a dinoflagellate sample with 1 L of sterile medium for washing. More, algal cell recovery becomes extremely difficult when the cells are stuck firmly to the filter, and the algal cells may be easily damaged during cell harvest. This is a very important consideration for fragile cells of the unarmored dinoflagellates as they are vulnerable to the shear force generated during filtration. Micropipetting method applied in *Coscinodiscus wailesii* was laborious, and a skilled operator was required to pipette the algal cells from a culture droplet and sequential wash in droplets^19^.

In the gradient centrifugation, the commonly used density media include Ficoll, Ludox and Percoll. Of these, Ficoll is not suitable for marine samples because it is a polysaccharide-based medium that is non-isotonic at high medium concentrations and becomes viscous liquid when dissolved in seawater^28,29^. Ludox and Percoll are both silicon-based density media compatible with seawater. Percoll is preferred for cell separation because it is less cytotoxic to the algal cells than is Ludox^30^. Few studies have demonstrated the feasibility of using Percoll density gradient centrifugation for microalgal samples^17,31^. Two fragile dinoflagellate species, *Heterosigma carterae* and *Cochlodinium polykrikoides*, have been efficiently harvested and recovered through centrifugation using 90% Percoll^17,31^.

### Bacterial removal by basic and extended protocols

In the present study, Percoll density gradient centrifugation was coupled with antibiotic treatment. It is because the use of antibiotics is one of the most common bacterial killing methods but antibiotics alone rarely achieve complete bacterial elimination from the algal culture^11^. Antibiotic susceptibility testing results in this study reveal that the bacteria associated with KMHK culture were sensitive to the antibiotic cocktail used, that is, a combination of 100 U of penicillin, 100 µg/mL streptomycin, 100 µg/mL gentamicin and 1 µg/mL tetracycline. Similarly, Ki and Han also reported that the combination of 100 mg/L streptomycin, 150 mg/L ampicillin, 150 mg/L penicillin G and 200 mg/L gentamicin effectively killed bacteria without having detrimental effects on the dinoflagellates *Peridinium bipes* and *A. tamarense*^12^. Guillard demonstrated that most algal species tolerated 100 mg/L penicillin, 25 mg/L streptomycin and 25 mg/L gentamicin reasonably well^32^. However, many red colonies, identified as those of *Rhodopirellula baltica* through 16 s rDNA sequencing analysis, were observed on the antibiotic susceptibility testing plate of KMHK cells containing only penicillin, streptomycin and gentamicin in our study. Tetracycline was therefore included in the present antibiotic treatment based on previous report that *Rhodopirellula* sp. was highly susceptible to 0.5 ppm of tetracycline^33^. It is common to modify the antibiotic cocktail for different algal cultures since the antibiotics depends on the microbiome. For instance, Su et al*.* treated *Alexandrium* cultures with a combination of gentamycin, streptomycin, cephalothin and rifampicin for 7 days to obtain axenic cultures^13^.

**Figure 3:**
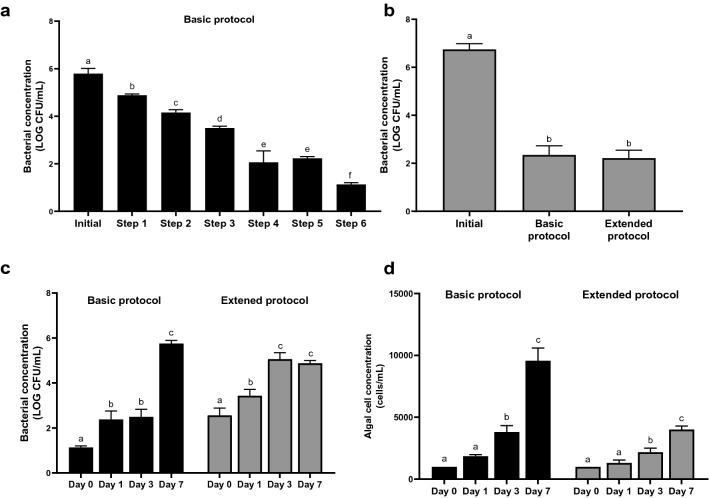
Bacterial removal in the KMHK sample using the basic and extended protocols. (**a**) Total bacterial count after different steps in the basic protocol. Initial: the initial bacterial count in KMHK samples at the beginning; Supplementary Fig. 1 illustrate the Step 1 to 6 of basic protocol. (**b**) Total bacterial count after the basic and extended protocols. Extended protocol refers to the descriptions in material and method. (**c**) Total bacterial count in the treated KMHK cultures at different days of cultivation. (**d**) Algal cell concentration during the regrowth of the treated KMHK cultures. All data are presented as mean ± standard deviations of three independent experiments (n = 3). Different letters on the top of the bar indicate that means were significantly different among samples at *p* ≤ 0.05 according to one-way analysis of variance followed by Tukey multiple comparison tests.

The effectiveness of the basic and extended protocols to remove bacteria in the algal samples is summarized in Fig. 3. The total bacterial count significantly reduced from initial 5.79 ± 0.22 log_10_ CFU/mL to 4.88 ± 0.05 log_10_ CFU/mL (*p* ≤ 0.05) after centrifuged with 90% Percoll (Step 1), even though the primary aim of this step was to condense the algal cells. This is probably due to the removal of numerous free-living bacteria in the supernatant, which was discarded. In the subsequent two gradient centrifugation using 90–50–30% density layers (Steps 2 and 3), approximately 12% of bacteria were further removed at *p* ≤ 0.05. The bacterial count did not show any additional reduction even when a step of a 90–50–30% density gradient centrifugation was added between Steps 3 and 4 (data not shown). After Step 4 with 48-h antibiotic treatment, the bacterial count was significantly reduced by 31% (Fig. 3a). These results indicated that numerous algae-associated bacteria could be effectively inhibited using the antibiotics but these steps could not completely eradicate the bacteria. No further decline in the bacterial count was found after Step 5 but decreased significant after Step 6, although both steps employed the same gradient centrifugation (90–50–30%). This could probably be the killing effect of the extended incubation of the bacteria with the remaining antibiotics, and/or the bacterial count significantly reduced by the gradient centrifugation in Step 5 was offset by the intracellular bacteria released from algal cell lysis during the antibiotic treatment. The effect of antibiotic exposure time on bacterial removal efficiency was shown in Table 1 below, while the existence of intracellular bacteria inside the dinoflagellate cells remains controversial^6^ and deserves more in-depth studies. The residue antibiotics, bacteria and algal cell debris were reported to suppress the algal cell growth^17,31^, these suppressions could be effectively removed in the present study as shown by algal regrowth (Fig. 3).

After all six steps of the present protocol, the total bacterial count remaining in the KMHK culture was 1.13 ± 0.07 log_10_ CFU/mL (equivalent to approximately 13 CFU/mL), indicating that > 99.9% of the bacteria were removed. Nevertheless, the bacterial count increased significantly to 5.75 ± 0.14 log_10_ CFU/mL (equivalent to approximately 5.86 × 10^5^ CFU/mL) in day 7 KMHK regrow culture after the protocol (Fig. 3c). We used the extended protocol to remove the remaining bacteria by repeating the 48-h antibiotic incubation and Steps 4–6 after the basic protocol (Fig. 3b). However, the total bacterial count did not have any significant change between the basic and extended protocols (Fig. 3b), demonstrating that the additional steps, that is, repeating the 48-h antibiotic incubation and Steps 4–6, in the extended protocol failed to eliminate the few remaining bacteria in the KMHK culture. Similar to the basic protocol, the remaining bacteria regrew significantly in day 3 and day 7 KMHK cultures after the extended one (Fig. 3c). More, the additional steps of antibiotic treatment and centrifugation even damaged the algal cells, as reflected by the regrowth of KMHK cells was strongly inhibited by the extended protocol (Fig. 3d). After the extended protocol, the KMHK cell density at day 7 was only half of that after the basic protocol that regrew normally and reached the cell density of approximately 10,000 cells/mL after 7 days, comparable to that of a routine culture. The algal growth rates (µ) after the basic and extended protocols were 1.29 and 1.14, respectively. This implied that the additional steps of antibiotic treatment in the extended protocol damaged the algal cells. It has been reported that antibiotics treatment could interfere the peptidoglycan biosynthesis and eventually inhibited chloroplast division^34^. The extended protocol was also ineffective in removing bacteria, probably due to limited bacterial removal capacity and/or insufficient dose and exposure time of antibiotics.

### Effect of initial algal cell density and antibiotic exposure times on bacterial removal

Bacterial cell concentration, antibiotic dose and antibiotic exposure time are critical factors affecting the efficiency of bacterial removal in algal samples. We hypothesized that the incomplete bacterial removal after the basic protocol was attributable to (1) the bacterial concentration in the algal culture exceeded the treatment capacity and (2) the dose and exposure time used in the antibiotic treatment were insufficient. To test insufficient dosing, a double antibiotic dose was used but there was no significant difference in the amount of bacterial removal between the normal and double doses of antibiotics used in the treatments (data not shown). Our observations also showed that the cell density of the 7-day KMHK culture treated with a double dose of antibiotics decreased from 10,000 to 489 cells/mL, clearly indicating a high antibiotic dose severely damaged the algal cells.

Both the amount and percentage of bacterial removal were independent of the initial algal cell density (Table 1). At least 94.49% of bacteria were removed in all treatments. With a 48-h antibiotic exposure time, the percentage of bacterial removal did not have any significant changes with increases of initial algal cell density, and were 94.49%, 99.84% and 99.93% at high, moderate and low densities, respectively. Similarly, no significant difference in the amount of bacterial removal (in terms of Log_10_ CFU/mL) was observed between low and moderate initial algal cell densities but were significantly higher than that at high initial algal cell density. With 96-h of antibiotic exposure, bacterial removal percentage was 100% at both high and low algal densities and 98.53% at moderate algal density. Similarly, bacterial removal of low and moderate initial algal cell densities were significantly higher than that at high initial algal cell density. These results indicated that the bacterial removal ability was independent of the initial algal cell density.

**Table 1:** Bacterial removal in the algal cultures with different initial algal cell densities and antibiotic exposure times in the basic protocol.

Initial algal cell density	Antibiotic exposure time (h)	Bacterial removal	Percentage of bacterial removal (%)	Bacterial regrowth in the treated algal culture*
		(Log_10_ CFU/mL)		
High	48	3.48 ± 0.1^a,A^	94.49 ± 5.02	Yes
	96	1.92 ± 0.03^b,A^	100.00 ± 0	No
Moderate	48	4.77 ± 0.1^a,B^	99.84 ± 0.15	Yes
	72	3.58 ± 0.09^b^	100.00 ± 0	Yes
	96	3.58 ± 0.09^b,B^	98.53 ± 2.54	Yes
Low	48	5.04 ± 0.15^a,B^	99.93 ± 0.07	Yes
	96	3.13 ± 0.16^b,C^	100.00 ± 0	No

Initial algal cell counts: High, 3.5 × 10^5^, Moderate, 1 × 10^5^ and Low, 3.5 × 10^4^ cells. Data of bacterial removal and percentage of bacterial removal are presented as means ± standard deviations of three independent experiments (n = 3).

*Samples were taken and monitored at days 1, 3, and 7 of the algal cultures. Different small and capital letters indicate that means were significantly different among antibiotics exposure times (a and b) and initial algal cell densities (A and B), respectively at *p* ≤ 0.05 according to one-way analysis of variance followed by Tukey multiple comparison tests.

When compare the antibiotic exposure time, bacterial removal with 48-h antibiotic treatment was significantly higher than that with other exposure times, regardless of the initial algal density (Table 1). A complete bacterial removal (100%) was detected under three conditions, that is, high and low initial densities with 96-h exposure time and moderate initial algal density with 72-h exposure time. The antibiotic exposure times ranging from several hours to 1 week have been reported in pervious researches, depending on the method used, and the bacterial cell count and composition in the algal sample^13,32^. The exposure time should be considered and controlled carefully because prolonged exposure to antibiotics may damage the algal cells and suppress their growth. However, the present study reveals antibiotic exposure time was not a critical factor for achieving 100% bacterial removal. On the other hand, the bacterial removal might be related to the bacterial cell count and composition present in the algal culture at the beginning. The microbiome can also change frequently during the routine cultivation of the algal cultures^35^.

Algal cells obtained from different protocols were then cultured for 7 days and then subjected to bacterial counting. A substantial number of bacteria was found in all treatments at the end of the algal regrow cultures, including the 72-h exposure achieving 100% bacterial removal, except two 96-h antibiotic exposure treatments (Table 1). Even in these two 96-h exposure treatments with 100% bacteria-free algal cultures, bacteria were detected after several generations of the algal culture (data not shown). These indicated that the axenicity of algal culture may not be guaranteed even when no bacteria are detected after treating the algal samples with our developed protocol.

The reappearance of bacterial growth may be due to the existence of a very small proportion of bacteria attached onto the algal cells after the protocol which was too little to be detected. Dinoflagellates are covered with complex and irregular cell surface structures^36^. Steric hindrance from parts of these algal surface areas may protect the firmly attached bacteria, making their detachment from the algal cells during gradient centrifugation difficult and decreasing antibiotic accessibility. Another possible explanation is that a few bacteria may have developed antibiotic resistance^11^, but this is unlikely in the present study as antibiotic resistance usually develops when the bacteria are continuously exposed to a nonlethal dose of an antibiotic^37^. Although why a few bacteria remained on the algal cells and regrew rapidly along with algal cell growth are poorly understood, it is necessary to have additional treatments such as serial dilution selection to ensure a true axenic algal culture is obtained.

### Selection of axenic algal cells through serial dilution

After 7 days of cultivation, KMHK cells from all dilutions described in Fig. 1 survived. No bacterial growth was observed in 42 KMHK cultures (out of total 45 cultures), although bacterial colonies were observed in one of the day 7 KMHK cultures (one culture in the 10^0^ dilution) and in another two of the day 21 KMHK cultures (one in 10^0^ dilution and one in 10^–1^ dilution) in the first trial (Supplementary Table 1). The results reiterate that a high proportion of the KMHK cells in the population is in fact axenic and the ratio of axenic to non-axenic clones in the algal cell population is high after the basic protocol, it is therefore highly feasible to obtain the axenic clones from the population through such serial dilution approach. Similar approaches have been reported in previous studies^5,12,38^. For instance, Ki and Han dispersed the algal cells in a 96-well plate after filtration and antibiotic treatment^12^. Sena et al*.* also serially diluted the cyanobacterial sample, *Arthrospira* spp., after antibiotics treatment^38^. Although the axenic clone could be obtained by plating the algal cells on agar^5^, this approach was not feasible for marine dinoflagellates because the cells were unable to grow on a solid medium.

### Verification of axenicity of algal cultures

**Figure 4:**
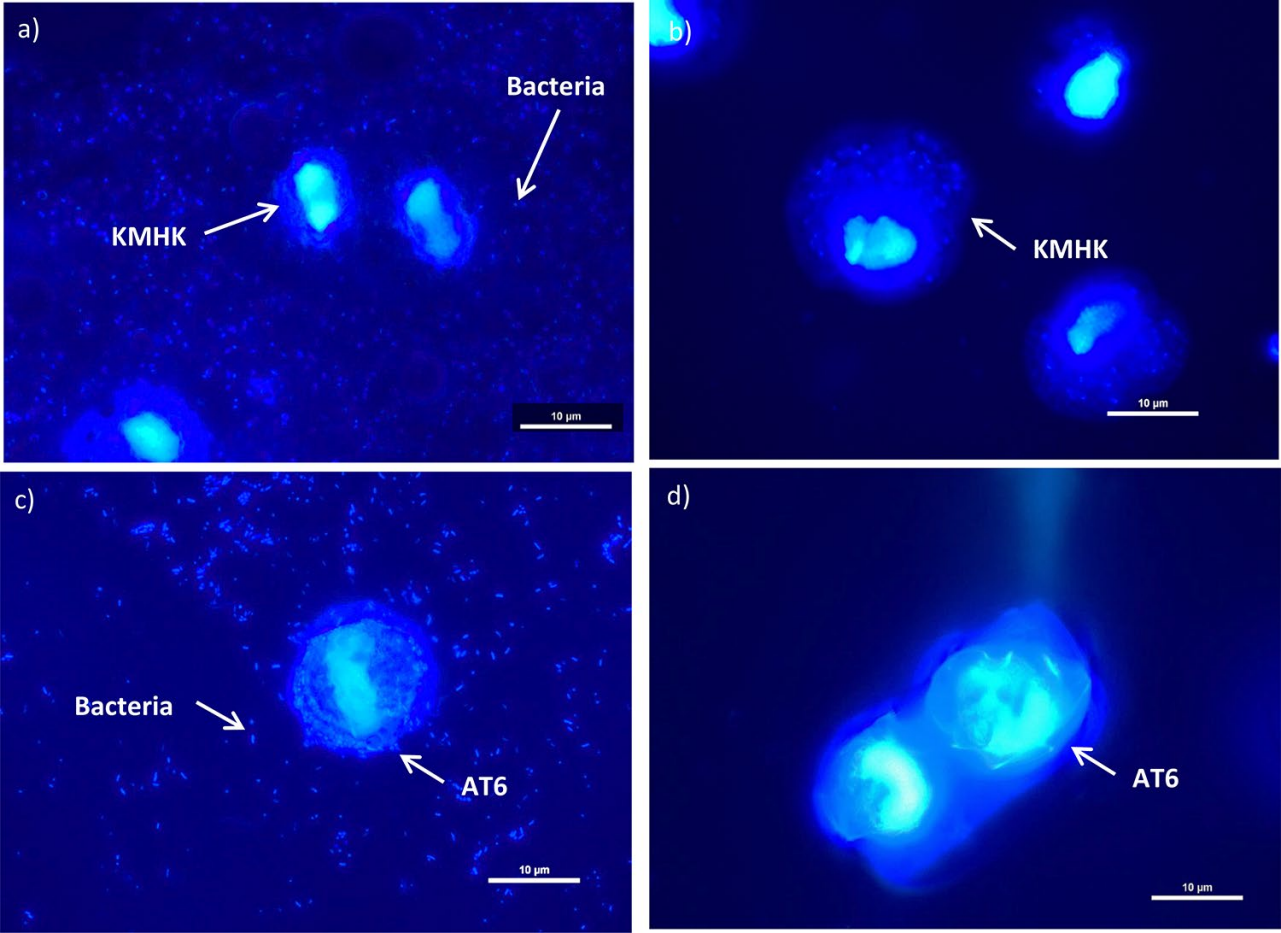
DAPI epifluorescence microscopic images of KMHK and AT6 samples under ×1000 magnification: (**a**) untreated (control) and (**b**) treated KMHK samples; (**c**) untreated (control) and (**d**) treated AT6 samples.

**Figure 5:**
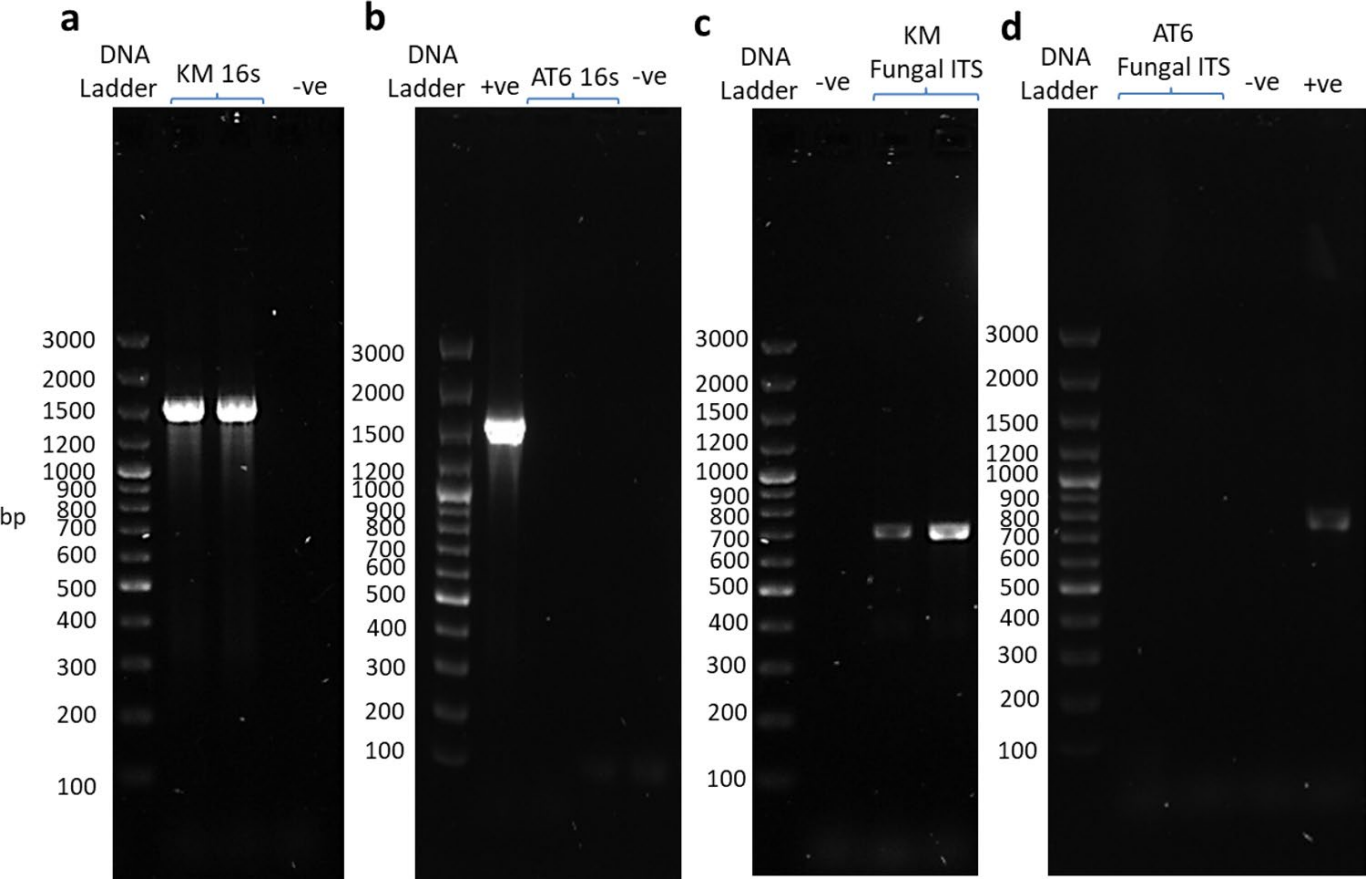
PCR amplification of bacterial 16 s rDNA for (**a**) KMHK and (**b**) AT6 samples and that of fungal ITS region for (**c**) KMHK and (**d**) AT6 samples obtained from basic protocol and serial dilution. +ve: positive control, -ve: negative control.

It has been reported that some marine bacteria grow very slowly on agar, something like 50 days, and some of them are unculturable^39,40^. Therefore, confirming the axenic state of the algal cultures is paramount. The axenic state of the two selected KMHK cultures was tested and results of DAPI epifluorescence microscopy show that no bacteria were observed in the treated KMHK cells (Fig. 4b) while bacteria were found in the untreated cells (Fig. 4a). For the rDNA sequencing analysis, a 1500-bp PCR product was obtained after bacterial 16S rDNA amplification (Fig. 5a). The BLAST of the sequence reveal that it shared 99.59% similarity with 16 s rDNA sequence in the plastid gene of *K. mikimotoi* (accession no. AB027236). Similarly, the 600-bp amplicon observed in the amplification of fungal ITS shared 99.67% similarity with the ITS sequence of *K. mikimotoi* (accession no. KT733616; Fig. 5c). These results confirm the absence of both culturable and unculturable bacteria and fungi in the treated KMHK cultures. It has been reported that the algal cultures must continually be treated with antibiotics in order to maintain their axenic status^15^, but algal cells may die after several sub-cultures because of prolonged antibiotic exposure. When this happens, it is usually too late to recover the cultures. In the present study, regular monitoring of the axenicity of the cultures was performed through bacterial colony counting, DAPI epifluorescence microscopy and rDNA sequence analysis. The established axenic cultures were maintained generations after generations without adding any antibiotics, and no bacteria were found in any of the sub-cultures being tested even after 30 generations (data not shown). The axenic cultures of KMHK were established successfully and maintained sustainably, indicating this methodology was a promising approach applicable to other unarmored dinoflagellates. To the best of our knowledge, this is the first successful establishment of an axenic culture for the unarmored dinoflagellate *K. mikimotoi*.

### Development of axenic culture of *A. tamarense* using our established methodology

**Figure 6:**
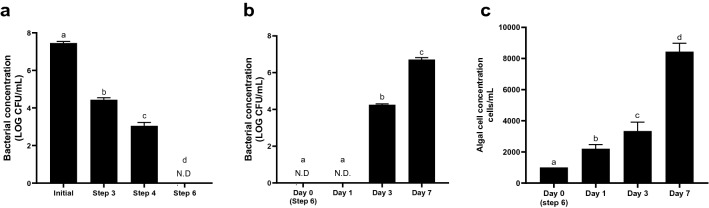
Bacterial removal in the *Alexandrium tamarense* (AT6) samples using the basic protocol. (**a**) Total bacterial count against different steps. Initial: the initial bacterial count present in AT6 at the beginning. (**b**) Total bacterial count in the AT6 culture obtained after the protocol at different days of cultivation. (**c**) Algal cell concentration during the regrowth of the AT6 culture obtained after the protocol. All data are presented as means ± standard deviations of three independent experiments (n = 3). N.D.: not detected. Different letters on the top of the bar indicate that means were significantly different among different samples at *p* ≤ 0.05 according to one-way analysis of variance followed by Tukey multiple comparison tests.

The established method was applied to obtain the axenic cultures of another dinoflagellate species, *A. tamarense* (AT6), a well-known paralytic shellfish toxin–producing agent which has been extensively studied in the past decades^41–44^. The AT6 culture with an initial bacterial count of 7.9 ± 0.08 log_10_ CFU/mL was subjected to the basic protocol and the total bacterial counts recorded after Steps 3, 4 and 6 were shown in Fig. 6a. The result was generally similar to that of KMHK culture using the basic protocol (Fig. 3a), except no bacteria were detected in the AT6 culture after Step 6. Even though 100% bacterial removal was achieved, bacterial regrowth was observed on days 3 and 7 of the treated AT6 culture (Fig. 6b). The bacterial count regrew significantly to 6.71 ± 0.08 log_10_ CFU/mL after 7 days of cultivation (Fig. 6c). The regrowth of bacteria from the treated AT6 culture achieving 100% bacterial removal confirmed that few bacteria attached at some points onto the algal surface were shielded. Biegala et al*.* reported that associated bacteria were attached onto the cell surface within the sulci and cingula of *A. tamarense*^6^.

For the serial dilution selection of AT6 cells after the basic protocol, culturable bacteria were observed in 11 of the 15 cultures in 10^0^ dilution (5/5 in trial 1; 4/5 in trial 2 and 2/5 in trial 3) after 7 days of algal cultivation. All these 15 cultures showed bacterial regrowth after 21 days of algal cultivation, but the number of cultures with bacterial regrowth decreased to 1 and 4 of the 15 cultures in 10^–1^ dilutions after 7 and 21 days of algal cultivation, respectively. In 10^−2^ dilutions, no culturable bacteria were observed in all the cultures after both 7 and 21 days of algal cultivation (Supplementary Table 1). These results further demonstrate the feasibility of using the stepwise serial dilution method to select axenic algae, and 10^–2^ dilutions offer the highest probability in acquiring the axenic clones. The bacterial status of two of these potential axenic AT6 cultures was further assessed through DAPI epifluorescence microscopy and bacterial rDNA and fungal ITS sequencing analysis. No bacteria were observed in the DAPI epifluorescence image of the treated AT6 cultures compared to the untreated control cultures (Figs. 4c,d). Neither bacterial 16 s rDNA band (Fig. 5b) nor fungal ITS region (Fig. 5d) was amplified in the treated AT6 samples. These results confirmed the axenic status of the AT6 cultures.

### Our established methodology

**Figure 7:**
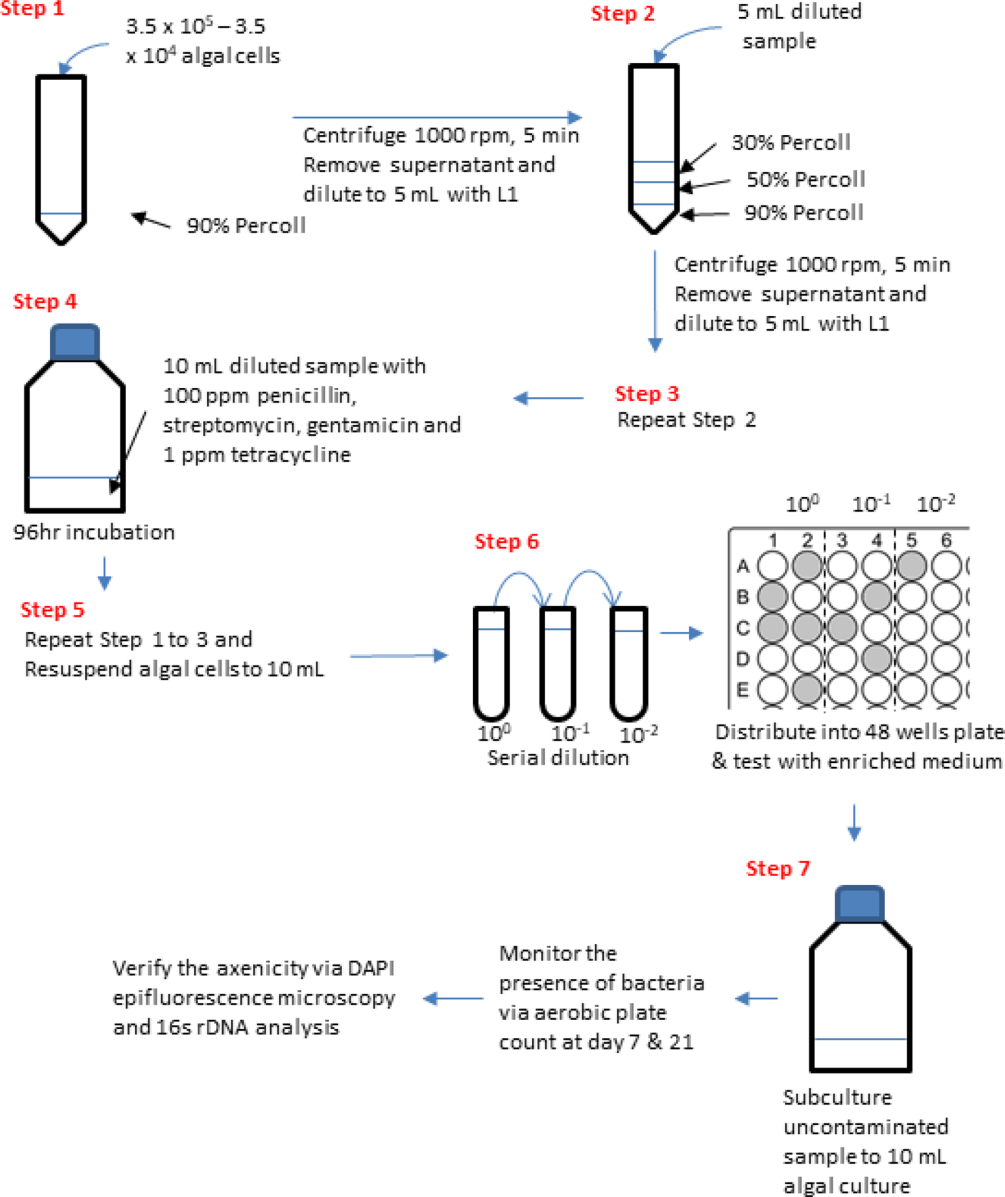
Workflow for establishing axenic cultures of dinoflagellate samples based on the approach demonstrated in this study.

The present results demonstrate the potential of our methodology to be used in the establishment of axenic cultures for both armored and unarmored dinoflagellates. Figure 7 summarizes the workflow and procedures of our methodology. This promising approach combines three techniques, Percoll density gradient centrifugation, antibiotic treatment and serial dilution. Density gradient centrifugation considerably reduces the bacterial population by the physical separation between the associated bacteria, mainly the free-living and loosely attached bacteria, and the dinoflagellate cells on the basis of cell size. Percoll density layers not only provide a matrix for separating the two types of cells effectively but also cushion the dinoflagellate cells against the impact of the mechanical force. The Percoll density layers together with the bactericidal action of the antibiotic treatment typically eradicate > 99% of the associated bacteria from the dinoflagellate culture. Our strategies not target at removing the remaining < 1% bacteria, but focus on differentiating and isolating the axenic dinoflagellate cells from those still bearing bacteria. The axenic clones are easily and effectively identified and selected through subsequent serial dilution. The cultures established by growing the selected clones are axenic, and their axenic status can be sustainably maintained for many generations without any antibiotic application. Apart from these two strains above, we have successfully established axenic cultures for other armored and unarmored species using our methodology, including *K. brevis* (CCMP 121), *Gymnodinium catenatum* (CCMP 1937), *Prorocentrum triestinum* (AD1), and *Amphidinium carterae* (CCMP 2281) as well as two other *K. mikimotoi* strains (CAWD133 and NIES2411) (data not shown).

Our approach has many advantages. First, all three techniques are inexpensive and straightforward. The entire procedure merely requires a benchtop centrifuge, incubators and algal cultivation chambers, without any need of expensive and complex equipment such as fluorescence-activated cell sorters^45^, inverted microscopes with micromanipulators^46^, and tailor-made microfluidics devices^47^. Second, unlike laborious techniques such as micro-pipetting^19,46,48^, the techniques involved in our approach are relatively straightforward and can be performed easily. Third, most of the steps could be completed within 2 h because of the simple procedure such as centrifugation, sub-culturing, bacterial count, etc. Notably, our method causes no damage to armored or unarmored dinoflagellate cells, which can be easily retrieved and re-cultivated after treatment. We believe that our method is applicable to all dinoflagellate cells, particularly species with fragile and vulnerable structures.

## Conclusions

The axenic cultures of *K. mikimotoi* and *A. tamarense* are successfully developed by combining Percoll density gradient centrifugation, antibiotic treatment and serial dilution techniques. This approach is inexpensive, straightforward, easy to perform and applicable to both armored and unarmored dinoflagellate species. The proposed workflow is efficient and promising for the establishment of axenic dinoflagellate cultures, a key prerequisite when researching the relationship between dinoflagellates and their associated bacteria.

## Supplementary Information

Supplementary Information.

Supplementary Information.

Supplementary Information.
