# Supplementary material for: Establish axenic cultures of armored and unarmored marine dinoflagellate species using density separation, antibacterial treatments and stepwise dilution selection
Source: Sci Rep. 2021 Jan 8;11:202. doi: 10.1038/s41598-020-80638-x (PMC7794416; doi:10.1038/s41598-020-80638-x)

**Establish Axenic Cultures of Armored and Unarmored Marine Dinoflagellate Species Using Density Separation, Antibacterial Treatments and Stepwise Dilution Selection**

Thomas Chun-Hung Lee^a^, Ping-Lung Chan^a^_,_ Nora Fung-Yee Tam^b^, Steven Jing-Liang Xu ^a^ and Fred Wang-Fat Lee ^a,^*

^a^ Department of Science, School of Science and Technology, The Open University of Hong Kong, 852, Hong Kong; [chhlee@ouhk.edu.hk](mailto:chhlee@ouhk.edu.hk); [plchan@ouhk.edu.hk](mailto:plchan@ouhk.edu.hk); [sjlxu@ouhk.edu.hk](mailto:sjlxu@ouhk.edu.hk); [wflee@ouhk.edu.hk](mailto:wflee@ouhk.edu.hk)

^b^ Department of Chemistry, City University of Hong Kong, 852, Hong Kong; [bhntam@cityu.edu.hk](mailto:bhntam@cityu.edu.hk)

*Correspondence: [wflee@ouhk.edu.hk](mailto:wflee@ouhk.edu.hk); +852 3120 2690.

**Supplementary Figure 1**. The procedure of the basic protocol


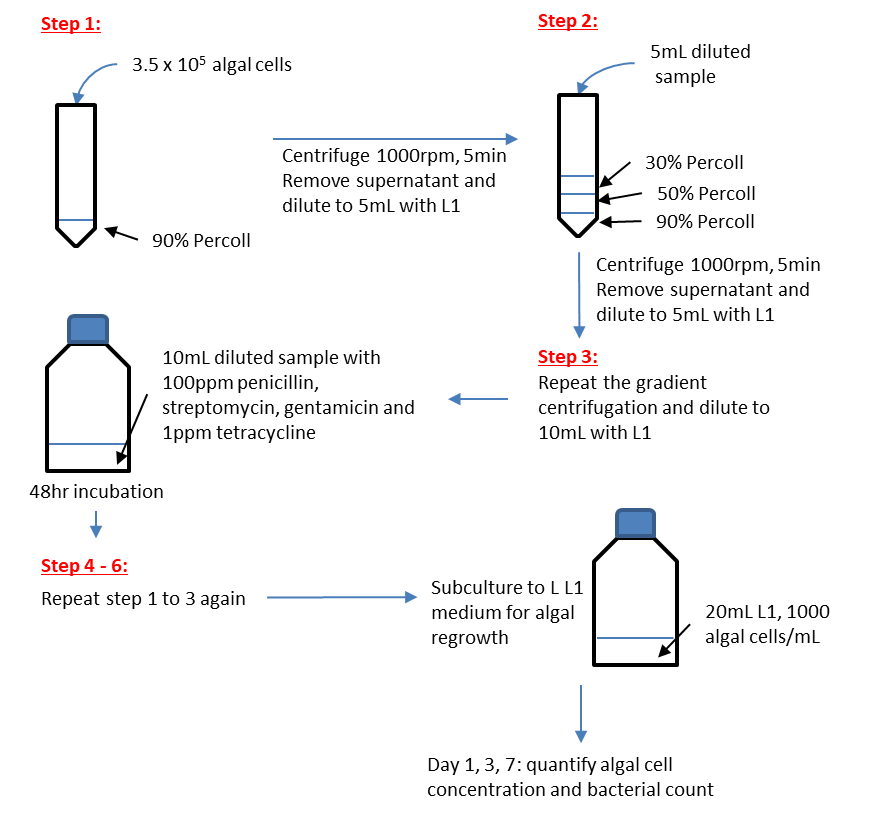

Supplement: Supplementary file 1 — Supplementary Information. [file 41598_2020_80638_MOESM1_ESM.docx]
