# Supplementary material for: Establish axenic cultures of armored and unarmored marine dinoflagellate species using density separation, antibacterial treatments and stepwise dilution selection
Source: Sci Rep. 2021 Jan 8;11:202. doi: 10.1038/s41598-020-80638-x (PMC7794416; doi:10.1038/s41598-020-80638-x)
Supplement: Supplementary file 3 — Supplementary Information. [file 41598_2020_80638_MOESM3_ESM.docx]

**Establish Axenic Cultures of Armored and Unarmored Marine Dinoflagellate Species Using Density Separation, Antibacterial Treatments and Stepwise Dilution Selection**

Thomas Chun-Hung Lee^a^, Ping-Lung Chan^a^_,_ Nora Fung-Yee Tam^b^, Steven Jing-Liang Xu ^a^ and Fred Wang-Fat Lee ^a,^*

^a^ Department of Science, School of Science and Technology, The Open University of Hong Kong, 852, Hong Kong; [chhlee@ouhk.edu.hk](mailto:chhlee@ouhk.edu.hk); [plchan@ouhk.edu.hk](mailto:plchan@ouhk.edu.hk); [sjlxu@ouhk.edu.hk](mailto:sjlxu@ouhk.edu.hk); [wflee@ouhk.edu.hk](mailto:wflee@ouhk.edu.hk)

^b^ Department of Chemistry, City University of Hong Kong, 852, Hong Kong; [bhntam@cityu.edu.hk](mailto:bhntam@cityu.edu.hk)

*Correspondence: [wflee@ouhk.edu.hk](mailto:wflee@ouhk.edu.hk); +852 3120 2690.

**Supplementary Table 1**. The bacterial regrowth on day 7 and 21 in (a) KMHK cells and (b) AT6 cells after serial dilution.

|  | | | | | | |
| --- | --- | --- | --- | --- | --- | --- |
| Day 7 | Trial 1 | Trial 2 | Trial 3 | Total | Mean in % | SD |
| 10^0^ | 1/5 | 0/5 | 0/5 | 1/15 | 7% | 12% |
| 10^-1^ | 0/5 | 0/5 | 0/5 | 0/15 | 0% | 0% |
| 10^-2^ | 0/5 | 0/5 | 0/5 | 0/15 | 0% | 0% |

| Day 21 | Trial 1 | Trial 2 | Trial 3 | | Total | | Mean in % | | SD |
| --- | --- | --- | --- | --- | --- | --- | --- | --- | --- |
| 10^0^ | 1/5 | 0/5 | 0/5 | | 1/15 | | 7% | | 12% |
| 10^-1^ | 1/5 | 0/5 | 0/5 | | 1/15 | | 7% | | 12% |
| 10^-2^ | 0/5 | 0/5 | 0/5 | | 0/15 | | 0% | | 0% |
| Total cultures with bacterial regrowth: | | | | 3/45 | |  | |  | |

|  | | | | | | |
| --- | --- | --- | --- | --- | --- | --- |
| Day 7 | Trial 1 | Trial 2 | Trial 3 | Total | Mean in % | SD |
| 10^0^ | 5/5 | 4/5 | 2/5 | 11/15 | 73% | 31% |
| 10^-1^ | 0/5 | 1/5 | 0/5 | 1/15 | 7% | 12% |
| 10^-2^ | 0/5 | 0/5 | 0/5 | 0/15 | 0% | 0% |

| Day 21 | Trial 1 | Trial 2 | Trial 3 | | Total | | Mean in % | | SD |
| --- | --- | --- | --- | --- | --- | --- | --- | --- | --- |
| 100 | 5/5 | 5/5 | 5/5 | | 15/15 | | 100% | | 0% |
| 10^-1^ | 1/5 | 3/5 | 0/5 | | 4/15 | | 27% | | 31% |
| 10^-2^ | 0/5 | 0/5 | 0/5 | | 0/15 | | 0% | | 0% |
| Total cultures with bacterial regrowth: | | | | 19/45 | |  | |  | |
